# A novel duplex real-time reverse transcriptase-polymerase chain reaction assay for the detection of hepatitis C viral RNA with armored RNA as internal control

**DOI:** 10.1186/1743-422X-7-117

**Published:** 2010-06-07

**Authors:** Shuang Meng, Jinming Li

**Affiliations:** 1 Graduate School, Peking Union Medical College, Chinese Academy of Medical Sciences, Beijing, China; 2 National Center for Clinical Laboratories, Beijing Hospital, Beijing, China

## Abstract

**Background:**

The hepatitis C virus (HCV) genome is extremely heterogeneous. Several HCV infections can not be detected using currently available commercial assays, probably because of mismatches between the template and primers/probes. By aligning the HCV sequences, we developed a duplex real-time reverse transcriptase-polymerase chain reaction (RT-PCR) assay using 2 sets of primers/probes and a specific armored RNA as internal control. The 2 detection probes were labelled with the same fluorophore, namely, 6-carboxyfluorescein (FAM), at the 5' end; these probes could mutually combine, improving the power of the test.

**Results:**

The limit of detection of the duplex primer/probe assay was 38.99 IU/ml. The sensitivity of the assay improved significantly, while the specificity was not affected. All HCV genotypes in the HCV RNA Genotype Panel for Nucleic Acid Amplification Techniques could be detected. In the testing of 109 serum samples, the performance of the duplex real-time RT-PCR assay was identical to that of the COBAS AmpliPrep (CAP)/COBAS TaqMan (CTM) assay and superior to 2 commercial HCV assay kits.

**Conclusions:**

The duplex real-time RT-PCR assay is an efficient and effective viral assay. It is comparable with the CAP/CTM assay with regard to the power of the test and is appropriate for blood-donor screening and laboratory diagnosis of HCV infection.

## Background

Hepatitis C virus (HCV) is one of the major causes of chronic liver diseases, which has infected an estimated 170 million people worldwide [1,2]. It is responsible for chronic liver diseases and is a risk factor for liver cirrhosis and hepatocellular carcinoma [3]. Early diagnosis and evaluation of HCV cases is very helpful for the management of the disease.

Since enzyme immunoassays have been used for blood-donor screening and laboratory diagnosis of HCV infection, a sharp decline has been observed in post-transfusion hepatitis C [4-6]. However, even with the most advanced third-generation assays, the HCV-antibody window period is approximately 58 days [7]. In addition, false-positive results may occur in patients with autoimmune diseases and in neonates born from mothers with chronic HCV infection [8-10]. Screening of HCV RNA by using nucleic acid amplification techniques (NATs) reduces the risk of HCV transmission and aids in the early detection of HCV infections [11]. Recently, assays based on real-time reverse transcriptase-polymerase chain reaction (RT-PCR) have been introduced in routine diagnostics and are rapidly replacing assays based on standard RT-PCR and signal amplification [12]. Unlike serological assays, those based on real-time RT-PCR can be used for the diagnosis of acute hepatitis before seroconversion and in the case of some seronegative patients with immune deficiency. Detection based on real-time RT-PCR is also useful for confirming indeterminate serological results and monitoring response to treatment [13].

The HCV genome is extremely heterogeneous. The reason for this genetic heterogeneity is the high error rate due to the lack of proofreading ability of the RNA-dependent RNA polymerase, which is responsible for the replication of the viral genome. Published sequence data indicate that the 5' untranslated region (UTR) is generally highly conserved among different HCV isolates [14] and is the target of most HCV assays. This region, however, also contains genotypically variable sequence positions, which allow discrimination of all the major types and many subtypes of HCV [15]. Several researchers have confirmed that nucleotide mutations and polymorphisms exist in the 5' UTR of the HCV genome [16-19]. Nucleic acid-based assays depend on hybridization between the template and PCR primers/probes [20], and mismatches can significantly reduce the viral detection and quantification efficiency. Thus, a single primer/probe, which is generally used in commercial HCV assays, may result in missing detections because of mismatches [12,21,22]. A duplex primer/probe assay can simultaneously amplify more than one target sequence [23,24]. Theoretically, some specimens are likely to be missed out on testing with a singleplex primer/probe assay but are detected by a duplex primer/probe assay. Some researchers have proved this by the use of multiple primer/probe sets, which significantly improved the performance of nucleic acid-based assays [25,26]. Sometimes, PCR inhibitors cannot be reliably removed from the sample and viral RNA may somewhat be degraded or may not be efficiently removed from the viral coat protein. Under these circumstances, internal controls (ICs), which are coextracted and coamplified with the viral RNA in the same reaction tube, can monitor the specimen extraction and amplification efficiency [27,28]. Thus, false-negative results can be avoided with the use of ICs.

In this study, we developed a duplex real-time RT-PCR assay using 2 sets of primers/probes and a specific armored RNA as IC. With the combination of the 2 sets of primers/probes, the performance of the assay was significantly improved, avoiding missing detections to the maximum possible extent.

## Results

### Optimal concentration of IC

Armored RNA was serially diluted and then spiked into the national reference material for HCV RNA (GBW09151; 2.26 × 10^2 ^IU/ml, 2.26 × 10^3 ^IU/ml, 3.97 × 10^4 ^IU/ml, 8.5 × 10^5 ^IU/ml). Armored RNA was coextracted and coamplified with the samples in the same reaction tube. According to the results presented in Table 1, 1000 copies/ml of armored RNA was used as the optimal concentration of IC in the HCV RNA duplex real-time RT-PCR assay.

**Table 1:** Optimization of the concentration of IC

Armored RNA concentration (copies/ml)	National reference material 4 for HCV RNA 8.5 × 10^5 ^IU/ml		National reference material 3 for HCV RNA 3.97 × 10^4 ^IU/ml		National reference material 2 for HCV RNA 2.26 × 10^3 ^IU/ml		National reference material 1 for HCV RNA 2.26 × 10^2 ^IU/ml		HCV 0 IU/ml
	
	IC (Cy5)Ct	HCV (FAM)Ct	IC (Cy5)Ct	HCV (FAM)Ct	IC (Cy5)Ct	HCV (FAM)Ct	IC (Cy5)Ct	HCV (FAM)Ct	IC (Cy5)Ct
100000	31.92	27.00	31.43	32.77	30.98	>45	30.43	>45	30.13
10000	36.51	26.93	35.39	31.20	34.58	36.29	34.29	41.55	34.21
1000	>45	26.44	38.35	30.14	37.72	33.23	36.68	36.67	36.47
0	>45	26.21	>45	30.11	>45	32.14	>45	36.12	>45

Concentrations of HCV RNA and armored RNA were indicated by FAM and Cy5 signals, respectively

### Intrinsic performance of the duplex real-time RT-PCR assay

#### Linearity

**Figure 1:**
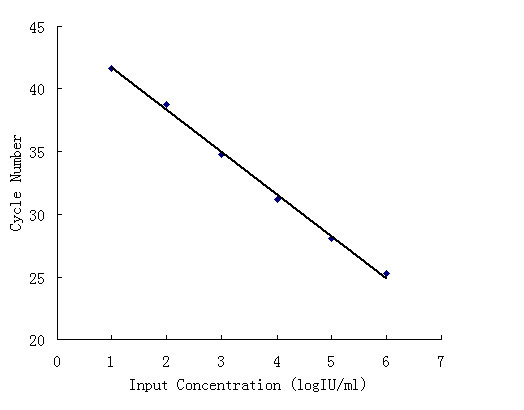
**Linearity of the duplex real-time RT-PCR assay**. Linearity of the duplex real-time RT-PCR assay was determined using serial 10-fold dilutions of a clinical sample at the following concentrations: 10, 10^2^, 10^3^, 10^4^, 10^5^, and 10^6 ^IU/ml. At each concentration, 3 replicates were tested in a single run. Linear relationship between the Ct values and the log_10 _HCV RNA concentration yielded R = 0.998.

Linearity of the duplex real-time RT-PCR assay was determined using serial 10-fold dilutions of a clinical sample at the following concentrations: 10, 10^2^, 10^3^, 10^4^, 10^5^, and 10^6 ^IU/ml. At each concentration, 3 replicates were tested in a single run. Liner regression analysis of the Ct values against the log_10 _HCV RNA concentration yielded R = 0.998 (Figure 1).

#### Sensitivity (LOD)

All HCV genotypes in the HCV RNA Genotype Panel for NATs (NIBSC, code 02/202, UK) could be detected by the duplex real-time RT-PCR assay. The proportion of positive results obtained from each input concentration was subjected to probit regression analysis (Table 2). The LOD of the duplex real-time RT-PCR assay was 38.99 IU/ml (95% confidence interval, 29.4-83.55 IU/ml).

**Table 2:** Limit of detection of the duplex real-time RT-PCR assay

HCV load (IU/ml)	Positive results/total tested	Positive results (%)
10^5^	24/24	100
10^4^	24/24	100
10^3^	24/24	100
10^2^	24/24	100
50	24/24	100
25	16/24	66.6
10	9/24	37.5

#### Specificity

The specificity of the duplex real-time RT-PCR assay was 100% in the testing of the HCV-negative serum samples.

#### Reproducibility

The intra-assay variation was assessed by testing 3 samples with different viral loads (10^5^, 10^4^, and 10^2 ^IU/ml) 10 times in a single run, while the inter-assay variation was assessed by testing the same samples 10 times in 10 separate runs. The intra-assay CV ranged from 0.93% to 1.34%, while the inter-assay CV ranged from 0.67% to 2.93% (Table 3).

**Table 3:** Reproducibility of the duplex real-time RT-PCR assay

Reproducibility	Target HCV RNA (IU/ml)	Number of determinations	Mean Ct	SD	CV (%)
Intra-assay	10^5^	10	27.15	0.25	0.93
	10^4^	10	31.09	0.33	1.07
	10^2^	10	38.31	0.51	1.34
Inter-assay	10^5^	10	27.05	0.29	1.08
	10^4^	10	31.05	0.21	0.67
	10^2^	10	37.23	1.09	2.93

### Comparison between singleplex primer/probe and duplex primer/probe real-time RT-PCR assays for HCV RNA detection

**Figure 2:**
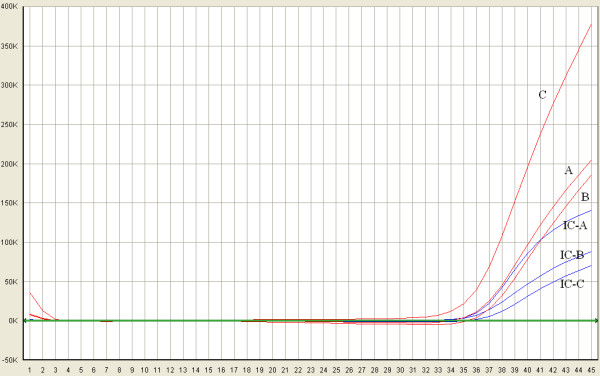
**Comparison of the duplex primer/probe and singleplex primer/probe assays**. The performances of the duplex primer/probe (C) and singleplex primer/probe (A, B) assays in the testing of the same serum sample obtained from a patient with low HCV viraemia were compared. The red amplification curves represent FAM fluorescence signal and the blue amplification curves represent Cy5 fluorescence signal. A: amplification plot of the HCV sample in the singleplex primer/probe A reaction system. B: amplification plot of the HCV sample in the singleplex primer/probe B reaction system. C: amplification plot of the HCV sample in the duplex primer/probe A and B reaction system. The Cy5 fluorescence signals indicate the amplification of IC. IC-A represents the amplification plot of ICs used in the singleplex primer/probe A reaction system. IC-B represents the amplification plot of ICs used in the singleplex primer/probe B reaction system. IC-C represents the amplification plot of ICs used in the duplex primer/probe A and B reaction system.

The specificity of these assays was 100%. Both singleplex primer/probe set A and set B failed to detect 1 serum sample. In contrast, the duplex primer/probe sets A+B detected all the 30 HCV-positive serum samples (data not shown). Figure 2 shows the performances of the duplex primer/probe (C) and singleplex primer/probe (A, B) assays in the testing of the same sample with of low load of HCV.

### Assay of 109 serum samples by using commercial kits

The results obtained by the duplex real-time RT-PCR assay were identical to those obtained by the CAP/CTM assay. BIOER and Kehua HCV RNA real-time RT-PCR assay kits failed to detect several samples, which were detected by the duplex real-time RT-PCR assay (Table 4).

**Table 4:** Testing results of different assays and kits for 109 serum samples

BIOER HCV real-time RT-PCR fluorescence detection kit	Kehua HCV RNA real-time RT-PCR detection kit	CAP/CTM assay	Duplex primer/probe assay	Number of samples detected
+	+	+	+	41
+	--	+	+	4
--	+	+	+	11
--	--	+	+	4
--	--	--	--	49

+: positive result, --: negative result

## Discussion

**Figure 3:**
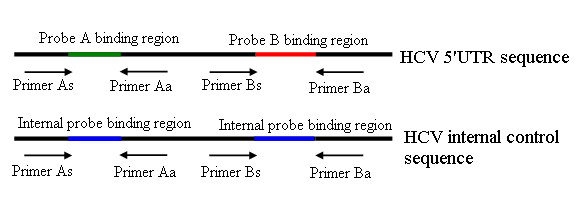
**Principles of the duplex real-time RT-PCR for detection of HCV RNA**. The assay was performed using 2 sets of primers/probes and a specific armored RNA as IC. Both the primer/probe sets A and B, in combination, detected the HCV 5' UTR sequence. Further, both the detection probes were labelled with the same fluorophore, i.e. FAM, at the 5' end and with the same quencher dye, i.e. Black Hole Quencher (BHQ), at the 3' end. The ICs had the same primer-/probe-binding sites and amplification efficiencies as the target nucleic acid but contained discriminating probe sequences.

In this study, all the 5' UTR sequences of HCV recorded in the Los Alamos National Laboratory HCV Sequence Database were aligned. The alignment results revealed several nucleotide polymorphisms in the 5' UTR. Thus, all HCV sequences cannot be detected by a singleplex primer/probe assay. In order to avoid missing detections because of mismatch between the template and PCR primers/probes, the duplex primer/probe assay was used. In this assay, 2 probes were labelled with the same fluorophore (FAM) at the 5' end; these probes could mutually combine, greatly improving the power of the test (Figure 3).

Compared with a singleplex primer/probe set, the duplex primer/probe set has many advantages. First, the duplex primer/probe set could detect all the HCV genotypes in the HCV RNA Genotype Panel for NATs and avoided missing detections to the maximum possible extent. Several assays using a singleplex primer/probe set produce false-negative results because of mismatches between the template and primers/probes [12,21,22,29]. This problem could be effectively resolved by using 2 sets of primers/probes, which has been proved in this study. The 2 sets of primers/probes could match interchangeably, improving the power of the test. For instance, Chevaliez et al. [30] reported the case of 2 patients infected with HCV genotype 4, whose serum samples with high viral load could not be detected by the CAP/CTM assay. Researchers found that the failing detections were probably related to nucleotide polymorphisms at positions 145 and 165. On the basis of the sequences of the 2 undetected HCV samples, we found that the 2 sets of primers/probes could detect these samples in theory, regardless of the nucleotide polymorphisms. Second, the duplex primer/probe assay can estimate the virus levels accurately. There are many reports about the underestimation of virus load by singleplex primer/probe assays [12,21,22,29,31]. For example, some patients with very low HCV viraemia may yield a negative result by the CAP/CTM assay [31]. The 2 sets of primers/probes used in our assay could match interchangeably, creating additional combinations with different primer-directed elongations. Figure 2 shows the performances of the duplex primer/probe and singleplex primer/probe assays in the testing of the same serum sample. Obviously, the fluorescence value of the 2 sets of primers/probes is higher than that of the single set of primers/probes, and cycle threshold (Ct) can shift towards left. As a result, the duplex primer/probe assay could strengthen the fluorescence signal of the low HCV viraemia samples and increase the probability of detection. Third, compared with two commercial HCV detection assays (BIOER and Kehua HCV fluorescence detection kits), the duplex primer/probe assay has many advantages. BIOER HCV fluorescence detection kit required 900-μl of serum for HCV RNA extraction. However, the duplex primer/probe assay barely needs 100-μl of serum for nucleic acid extraction. The latter has more wide range of application, especially in the case of fewness of sample. Moreover, the duplex primer/probe assay has lower LOD (38.99 IU/ml) than BIOER and Kehua HCV fluorescence detection kits, and the cost of the duplex real-time RT-PCR assay was lower than that of the two commercial HCV detection assays. Fourth, the sensitivity of the duplex primer/probe assay is high and can be compared with that of the CAP/CTM assay. In the CAP/CTM assay, HCV RNA was extracted from 850-μl serum and then eluted with 65-μl of elution buffer. Finally, 50-μl extract was used as the template in 100-μl reaction volume [32]. In the duplex primer/probe assay, HCV RNA was extracted from 100-μl serum and then eluted with 20-μl of diethyl pyrocarbonate-treated H_2_O. Finally, 10-μl extract was used as the template in 25-μl reaction volume. The LOD of the duplex primer/probe assay was 38.99 IU/ml, which is higher than that of the CAP/CTM assay (15.0 IU/ml). Considering that HCV RNA was extracted from 850-μl serum and the reaction volume increased to 100-μl, we believed that the LOD of the duplex primer/probe assay could be comparable with or even exceed that of the CAP/CTM assay.

In this study, armored RNA was successfully used as IC in the duplex real-time RT-PCR assay. The IC spiked into the specimens could monitor the specimen extraction and amplification efficiency, saving additional labour-intensive procedures and expenditure of costly external control reagents. ICs include noncompetitive ICs and competitive ICs (CICs). In the noncompetitive IC strategy, separate primer pairs are used to detect ICs and the target nucleic acids. In a previous study, the performance of noncompetitive ICs was not perfect for the target nucleic acids because of differences in the amplification efficiencies [33]. In our study, CICs were constructed, which hybridized with the same primers and had identical amplification efficiencies as the target nucleic acid but contained discriminating probe-binding sequences [34]. In order to avoid the suppression of target amplification, the concentration of armored RNA spiked into the samples was optimized. According to the results shown in Table 1, 1000 copies/ml of armored RNA was used as the optimal concentration in the duplex real-time RT-PCR assay.

In the 109 serum samples collected from Shenzhen Blood Center, the prevalent genotypes of HCV should be 1 and 2 [35]. In the study of Chevaliez et al. [30], the CAP/CTM assay failed to detect HCV genotype 4. Thus, the testing results of the duplex real-time RT-PCR for the 109 serum samples, which were identical to those of the CAP/CTM assay, should be correct. The LOD of the duplex real-time RT-PCR assay was 38.99 IU/ml and the specificity was 100%. Furthermore, the cost of the duplex real-time RT-PCR assay was considerably lower than that of the CAP/CTM assay, and hence, the former assay is more suitable for large-scale use.

## Conclusions

The duplex real-time RT-PCR assay is comparable with the CAP/CTM assay with regard to the power of the test and is appropriate for blood-donor screening and laboratory diagnosis of HCV infection.

## Materials and methods

### Standards

A dilution series of the World Health Organization (WHO) Second International Standard for HCV RNA (National Institute for Biological Standards and Control (NIBSC), code 96/798, UK) was used to determine the limit of detection (LOD) of the duplex real-time RT-PCR assay at the following concentrations: 10, 25, 50, 10^2^, 10^3^, 10^4^, and 10^5 ^IU/ml. Each dilution of the WHO Standard was tested in a batch of 4 replicates in 6 separate runs, i.e. for each dilution, a total of 24 replicates were tested.

Linearity of the duplex real-time RT-PCR assay was determined using serial 10-fold dilutions of a clinical sample at the following concentrations: 10, 10^2^, 10^3^, 10^4^, 10^5^, and 10^6 ^IU/ml. At each concentration, 3 replicates were tested in a single run.

Inter-assay and intra-assay variations were calculated using a set of 3 samples with different viral loads (10^5^, 10^4^, and 10^2 ^IU/ml), which were tested 10 times in 3 different assays on different days.

The HCV RNA Genotype Panel for NATs (NIBSC, code 02/202, UK) was used to assess the performance of the duplex real-time RT-PCR assay.

### Patient serum samples

A total of 109 serum samples were collected from Shenzhen Blood Center (Guangdong, China). Each sample was divided into 4 aliquots and frozen to -80°C within 2 h of receiving [36]. These samples were used to compare the performances of BIOER HCV real-time RT-PCR fluorescence detection kit (Hangzhou BIOER Technology Co. Ltd., Hangzhou, China), Kehua HCV RNA real-time RT-PCR detection kit (Shanghai Kehua Bio-Engineering Co. Ltd., Shanghai, China), qualitative duplex real-time RT-PCR assay, and COBAS AmpliPrep (CAP)/COBAS TaqMan (CTM) assay (Roche Molecular Systems, Pleasanton, CA).

A total of 100 HCV-negative serum samples were obtained from blood donors, including those with hepatitis A, hepatitis B, hepatitis E, human immunodeficiency virus type 1 infection, and human T-cell leukaemia virus infection (confirmed at the blood bank), and negative serum samples obtained from normal persons were used for determining the specificity of the duplex real-time RT-PCR assay.

Further, 40 HCV serum samples were collected from Beijing Blood Center (Beijing, China); the samples included 30 HCV-positive and 10 HCV-negative samples (confirmed at the centre). These samples were used for comparing the performances of singleplex primer/probe and duplex primer/probe assays.

### Primer/probe design

HCV sequences were aligned using sequence comparison software. Based on the consensus sequences of the HCV genome, 2 sets of primers/probes were designed, which, in combination, could detect all the HCV sequences recorded in the Los Alamos National Laboratory HCV Sequence Database [37]. Probes for the detection of HCV and IC were labelled with 6-carboxyfluorescein (FAM) and a cyanine dye, Cy5, at the 5' end, respectively (Table 5).

**Table 5:** Primers/probes used in the study

Primer/probe	Primer/probe sequence (5'--3')	Position
As	5'-GAGTAGTGTTGGGTCGCGAA-3'	256--275
Aa	5'-GTGCACGGTCTACGAGACCTC-3'	320--340
Ap	FAM5'-CCTGATAGGGTGCTTGCGAGTGCC-3' BHQ	292--315
Bs	5'-AGCGTCTAGCCATGGCGTTAGTAT-3'	74--97
Ba	5'-TCCTCGCAATTCCGGTGTACTC-3'	161--182
Bp	FAM5'-CCCCCCTCCCGGGAGAGCCATAGT-3' BHQ	121--144
ICp	Cy55'-TTCCGCTGCCTGCTCAGTCGATCC-3' BHQ	

BHQ: Black Hole Quencher dye

### Construction of IC

**Figure 4:**
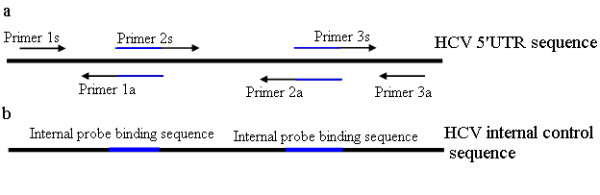
**Construction of IC by using overlap extension PCR**. (a) The internal probe-binding sequences were introduced into the HCV 5' UTR sequence by 3 cycles of PCR using primers designed by amplifying overlapping regions. (b) Constructed IC sequence. The blue portion represents the internal probe-binding sites.

IC sequences were identical to the wild-type HCV sequences, except for the probe Ap- and probe Bp-binding site sequences, which were replaced by the internal probe sequences (Figure 3). Gene splicing by overlap extension PCR was performed to construct an IC sequence containing 3 fragments (Figure 4). The overlap extension PCR product was cloned into the plasmid pACYC-MS2 [38] (constructed at our laboratory) and then verified by sequencing. The plasmids pACYC-MS2-IC were transformed into competent *Escherichia coli *BL21 (DE3) strains. After expression and purification, the armored RNA was harvested and quantified.

In order to determine the optimal concentration of IC used in the duplex real-time RT-PCR assay, the armored RNA was serially diluted and then spiked into the national reference material for HCV RNA (GBW09151; 2.26 × 10^2 ^IU/ml, 2.26 × 10^3 ^IU/ml, 3.97 × 10^4 ^IU/ml, 8.5 × 10^5 ^IU/ml). Thereafter, it was coextracted and coamplified with the samples in the same reaction tube.

### Nucleic acid extraction

RNA was extracted from 0.1-ml sample by using extraction reagents of the Kehua HCV RNA real-time RT-PCR detection kit (Shanghai Kehua Bio-Engineering Co. Ltd.) according to the manufacturer's instructions. The extracted RNA was eluted in 20 μl of diethyl pyrocarbonate-treated H_2_O and used as the template for the duplex real-time RT-PCR assay.

### Duplex real-time RT-PCR amplification for HCV RNA detection

The duplex real-time RT-PCR assay was performed on the ABI PRISM system (Applied Biosystems, America) by using 10 μl of RNA (using extraction reagents of the Kehua HCV RNA real-time RT-PCR detection kit) in a 25-μl volume containing 12.5 μl of 2× QuantiTect Probe RT-PCR Master Mix and 0.25 μl of QuantiTect RT Mix (QIAGEN, German). In the singleplex mode, either the primer/probe set A or the primer/probe set B was used in the reaction, while in the duplex mode, both the primer/probe sets A and B were used in RT-PCR. Armored RNA particles, added to each sample prior to extraction, were used as ICs in the extraction and amplification processes.

### Comparison between singleplex primer/probe and duplex primer/probe real-time RT-PCR assays for HCV RNA detection

The 40 serum samples collected from Beijing Blood Center were tested by singleplex primer/probe and duplex primer/probe assays, and the results were then compared.

### Commercial kits for HCV RNA detection

A total of 109 serum samples were tested using BIOER HCV real-time RT-PCR fluorescence detection kit (Hangzhou BIOER Technology Co. Ltd.), Kehua HCV RNA real-time RT-PCR detection kit (Shanghai Kehua Bio-Engineering Co. Ltd.), and CAP/CTM assay kit. All the operation steps were carried out according to the instructions given in the manuals provided by the manufacturers.

**(i) Detection using BIOER HCV real-time RT-PCR fluorescence detection kit**. HCV RNA was recovered from 900-μl of serum and quantified in the LineGene real time PCR assay system, according to the manufacturer's instructions. The results were determined based on the Ct values. The LOD of BIOER HCV fluorescence detection kit was 500 IU/ml.

**(ii) Detection using Kehua HCV RNA real-time RT-PCR detection kit**. HCV RNA was extracted from 100-μl sample and eluted in 20-μl of diethyl pyrocarbonate-treated H_2_O. 12.5-μl extract was used as the template in 25-μl reaction. RT-PCR was carried out in a 32-well Lightcycler thermal cycles system (Roche). The LOD of Kehua HCV RNA assay kit was 500 IU/ml.

**(iii) Detection using CAP/CTM HCV assay kit**. The CAP/CTM test utilizes automated specimen preparation on the COBAS AmpliPrep Instrument by a generic silica-based capture technique. HCV RNA was extracted from 850-μl serum and then eluted with 65-μl of elution buffer. Finally, 50-μl extract was used as the template in 100-μl reaction volume. The Cobas TaqMan 48 Analyzer was used for automated real-time RT-PCR amplification and detection of PCR products, simultaneously. HCV RNA levels were expressed in IU/ml. The LOD of CAP/CTM HCV assay was 15 IU/ml.

### Data analysis

Results are expressed as mean and standard deviation (SD), as appropriate. The intra-assay and inter-assay variations are expressed as SD and coefficient of variation (CV), based on the mean Ct values. Probit analysis was performed to determine the LOD. The LOD was determined as 95% probability of obtaining a positive HCV RNA result. Correlation coefficients (R) were calculated for linearity data.

## Competing interests

The authors declare that they have no competing interests.

## Authors' contributions

SM planned the experimental design and drafted the manuscript. JL conceived the study, participated in its design and coordination, and helped to revise the manuscript. All authors read and approved the final manuscript.
